# Triaging Clients at Risk of Disengagement from HIV Care: Application of a Predictive Model to Clinical Trial Data in South Africa

**DOI:** 10.2147/RMHP.S510666

**Published:** 2025-05-16

**Authors:** Mhairi Maskew, Shantelle Parrott, Lucien De Voux, Kieran Sharpey-Schafer, Thomas Crompton, Ashley Christopher Govender, Pedro Terrence Pisa, Sydney Rosen

**Affiliations:** 1 Health Economics and Epidemiology Research Office, Faculty of Health Sciences, University of the Witwatersrand, Johannesburg, South Africa; 2 Palindrome Data, Cape Town, South Africa; 3 Right to Care, Strategic Information and Data Science Unit, Johannesburg, South Africa; 4 Department of Human Nutrition and Dietetics, Faculty of Health Sciences, University of Pretoria, Pretoria, South Africa; 5 Department of Global Health, Boston University School of Public Health, Boston University, Boston, MA, USA

**Keywords:** HIV service delivery, retention, risk triaging, machine learning, predictive modelling

## Abstract

**Purpose:**

To reach South Africa’s targets for HIV treatment and viral suppression, retention on antiretroviral therapy (ART) must increase. Here, we aim to successfully identify ART clients at risk of loss from care prior to disengagement.

**Patients and Methods:**

We applied a previously developed machine learning and predictive modelling algorithm (PREDICT) to ART client data from SLATE I and II trials. The primary outcome was interruption in treatment (IIT), defined as missing the next scheduled clinic visit by >28 days. We tested two risk triaging approaches: 1) threshold approach classifying individuals into low, moderate, or high risk of IIT; and 2) archetype approach identifying subgroups with characteristics associated with risk of ITT. We report associations between risk category groups and subsequent IIT at the next scheduled visit using crude risk differences and relative risks with 95% confidence intervals.

**Results:**

SLATE datasets included 7199 client visits for 1193 clients over ≤14 months of follow-up. The threshold approach consistently and accurately assigned levels of IIT risk for multiple stages of the care cascade. The archetype approach identified several subgroups at increased risk of IIT, including those late to previous appointments, returning after a period of disengagement, living alone or without a treatment supporter. Behavioural elements of the archetypes tended to drive the risk of treatment interruption more consistently than demographics; eg adolescent boys/young men who attended visits on time experienced the lowest rates of treatment interruption (10% PREDICT datasets; 7% SLATE datasets), while adolescent boys/young men returning after previously disengaging had the highest rates of subsequent treatment interruption (31% PREDICT datasets; 40% SLATE datasets).

**Conclusion:**

Routinely collected medical record data can be combined with basic demographic and socioeconomic data to assess individual risk of future treatment disengagement. This approach offers an opportunity to prevent disengagement from HIV care, rather than responding only after it has occurred.

**Trial Registration:**

SLATE I trial: Clinicaltrials.gov NCT02891135, registered September 1, 2016. First participant enrolled March 6, 2017, in South Africa and July 13, 2017, in Kenya. SLATE II trial: Clinicaltrials.gov NCT03315013, registered 19 October 2017. First participant enrolled 14 March 2018.

## Introduction

With the successful expansion of universal access to HIV treatment around the world, retaining persons living with HIV in lifelong antiretroviral therapy (ART) has emerged as one of the most important challenges to HIV epidemic control.^1^ In South Africa, a country with one of the world’s largest populations on ART, an estimated 30–40% of clients disengage from HIV care within 6 months of initiating ART.^2^,^3^ Disengagement is a complex, multi-faceted problem and several factors associated with disengagement have been described including individual, interpersonal, health system, and societal or structural factors.^4^ For those who disengage from care (ie are not retained), the most common intervention continues to be after-the-fact tracking and tracing efforts, in which healthcare workers attempt to contact disengaged ART clients and encourage and/or assist them to return to care. These efforts have had mixed results, in terms of achieving re-engagement in care.^5–11^

A major drawback to all tracking and tracing programs is that they can only intervene after a person disengages from care. Little is done to distinguish those at higher risk of dropping out of care in advance, before disengagement occurs. Instead, the same advance support is offered to all, regardless of risk level. A strategy for identifying individuals at high risk of disengagement before they interrupt care would allow interventions to be targeted to those in need up front, before any damage is done while conserving the resources that might otherwise be expended on low-risk clients who require little or no intervention to remain in care.^12^ To put such a strategy into practice, both accurate pre-interruption risk triaging and a practical, low-cost tool that frontline healthcare workers can use to identify ART clients for differing levels of retention support and interventions are needed.

A number of previous efforts have been made to predict risks of poor outcomes among people living with HIV.^13–19^ While several models include basic demographic characteristics such as age and sex and clinical history such as baseline CD4 count to predict risk, the mechanisms driving risk within demographic subgroups at higher risk of disengagement than their age/sex peers remain unclear. Other characteristics that predict risk may be important to identify because within virtually any “risky” age/sex stratum, such as young men,^20^,^21^ a majority of individuals remain low risk and achieve good outcomes without intervention. In a 2018 population survey in KwaZulu Natal, South Africa, for example, young men aged 15–29 were the highest risk age/sex group identified, but more than half of them (51.5%) were virally suppressed.^22^ Yet, few approaches to risk prediction have progressed beyond the derivation of a risk score or model.^23^ Among those that have been implemented in clinical settings to predict outcomes such as risk of HIV acquisition,^24^ virologic failure, and treatment interruption,^25^,^26^ none has been successfully scaled at the national level. In addition, the accuracy of risk prediction models increases when data on prior treatment interruptions are included in the model (those who have previously disengaged from care are the most likely to experience another treatment interruption assuming the barrier to continuity of care has not been addressed).^27^ This presents a conundrum for risk triaging approaches – we need tools that identify clients at risk of disengaging from care prior to that event occurring, but a risk model’s ability to discriminate high- from low-risk clients may be driven by knowledge of a prior treatment interruption.

We previously applied machine learning and predictive algorithms^27^ to routinely collected longitudinal HIV phenotypic and clinical outcome data from the South African HIV treatment programme, one of the largest globally.^28^ The PREDICT model aimed to identify those at risk of a near-term interruption in treatment (IIT), defined as missing their next scheduled clinic visit by more than 28 days. To move beyond the age/sex and visit history characteristics that are currently routinely collected in electronic medical records, we reproduced this model in a smaller South African HIV clinical trial dataset from the SLATE trials^18^,^29^ containing socioeconomic indicators. We then utilized the output for two risk score triaging approaches to identify those at risk for disengagement from care: 1) a threshold approach to segment populations into risk groups; and 2) a series of archetypes characterizing social and behavioral client profiles. Here, we describe the development of these approaches and estimate associations with risk of disengagement from care, providing the basis for future development of a practical, point-of-care risk triaging tool.

## Materials and Methods

### Population and Data Sources

The two approaches to risk triaging were developed using output derived from two machine learning models. The first, PREDICT (Prioritizing Retention Efforts using Data Intelligence and Cohort Targeting),^27^ was initially trained and tested on routinely collected, anonymized, longitudinal medical record data from clients accessing HIV care and treatment at public sector treatment sites in Mpumalanga and the Free State between January 2016 and December 2018. These records contain information on clients’ clinical and antiretroviral treatment histories, including scheduled and attended clinical visits, laboratory test results, and basic demographic characteristics (age and sex). On average, PREDICT correctly identified two out of three clients who missed their next scheduled clinic visit. The model was recently validated in a different population and geographic setting in South Africa and demonstrated almost identical performance metrics.^30^

For the second model, the SLATE model, we used client survey and medical record data collected for the SLATE I and SLATE II trials, which were randomized evaluations of a clinical algorithm to determine eligibility for same-day initiation of ART at three primary healthcare facilities in Gauteng Province.^18^,^29^,^31^ SLATE enrolled non-pregnant adults who presented at the study clinics for any kind of HIV care, including diagnosis, and were not yet on ART. Participants completed a baseline survey that included demographic and socioeconomic characteristics, HIV testing and treatment history, and social indicators including disclosure status. Participants were then passively followed up for 14 months after study enrolment through clinic medical records observing scheduled and attended clinic visits at the study sites.

### Study Outcomes

The primary outcome of interest was retention in HIV care. We considered a client to be retained in care if a clinic visit was observed before or within 28 days of the next scheduled appointment date in that client’s medical record.^2^ Conversely, we defined a client to have experienced an interruption in treatment (IIT) when a client did not attend a clinic visit within 28 days of their scheduled appointment. This 28-day threshold was selected to align with the US President’s Emergency Program for AIDS Relief (PEPFAR) guidelines^32^ and with South Africa’s 2023 Adherence Guidelines for HIV, TB and NCDs.^33^ We restricted the analysis to visits scheduled a minimum of three months prior to the database censor date to allow for one month to meet the outcome definition and a further two months to allow for capturing visit data into the EMR. All raw data available in the source datasets were considered as potential predictors of IIT. These included de-identified data characterizing client demographics, HIV testing and ART treatment history, socio-economic indicators (employment, income), disclosure, drug regimen data, visit history and patterns, and ART monitoring laboratory test results.

### Model Building and Performance

Both the PREDICT and SLATE models used the AdaBoost (adaptive boosting binary classification) algorithm from scikit-learn.^34^ The model building and validation process is detailed in Supplementary Table 1 and also described elsewhere.^27^,^30^ In short, each of the source datasets is split into training and test sets. Training sets are datasets with known exposure and outcome variables used in machine learning approaches to allow the algorithm to “learn” the predictive importance of exposure variables in terms of correctly classifying each specified outcome. For test sets, the exposure variables are separated from the outcome variables (unseen) and given to the final classifier algorithm. The datasets originally had an imbalanced outcome variable (rate of IIT 11% in PREDICT and 14% in SLATE), which could lead to algorithm bias where model predictions favour the majority class (on-time visits). To address this, the training process included random down sampling (PREDICT) and up sampling (SLATE) the majority class to match the minority class size. The model is then tested on an unseen subset of data from its respective dataset set by generating predicted outcomes for each observed visit using the predictor variables from the unseen test set. In this way, the model produces an overall predicted risk score for each visit that indicates the likelihood that the next scheduled visit will not be attended on time and will be classified as an interruption in treatment. These predicted outcomes are then compared to the known outcomes in the test set, and the model is scored according to standard test performance metrics (sensitivity, specificity, positive and negative predictive value and area under the curve). To avoid overfitting, we included random sampling across multiple runs and splits of the train/test sets. Furthermore, for PREDICT, we kept a hold-out set of the most recent four months of visits after the original training and test period, to simulate how the model would perform in those conditions. In this analysis, to determine the value of the additional variables added to the medical record data using the SLATE baseline survey questionnaires, we estimated model performance metrics when restricting the SLATE model to the set of variables that were available to the PREDICT model (ie, data from routinely collected medical records only).

Finally, we used traditional resampling techniques including random bootstrap resampling and cross-validation to assess the stability of factors like disclosure, employment, and home support as risk factors for ART retention. We conducted sensitivity checks across demographic groups to see where the signal was general or specific and tested alternative model specifications like logistic regressions and different binary classifiers. We also conducted stakeholder consultations with healthcare providers, social workers, and community health workers to explore where models aligned with their experience.

### Risk Score Triaging

We next adopted two approaches to create a risk score triaging system to identify groups at risk of IIT. Output from both the SLATE and PREDICT models were used in each of the two risk-score triaging systems and are presented stratified by the source model for comparison throughout, with the exception of situations where the model did not contain variables required to classify risk groups or profiles. The two approaches are described below and compared in Table 1.

**Table 1:** Comparison of Threshold and Archetype Approaches to Risk Triaging

Property	Threshold Approach	Archetype Approach
General approach	Classifies clients into groups at low (green), moderate (yellow) or high (red) risk of outcome based on predictive model scores	Identifies sub-clusters of client populations with characteristics associated with risk of outcome
Automation	Can be automated into predictive model output; integrated into EMR	Currently involves manual application of clustering characteristics identified by machine learning model. Automation through EMR integration may be possible in future versions
Applicability	Applicable to any dataset and population	Specific to population context and characteristics
Intuitiveness or understandability	Requires score to be calculated beforehand and may not always be consistent with clinician assessment of risk	Intuitive, easy to apply with clients at point of care
Identification of drivers of risk	Mechanism behind risk not readily apparent	Some underlying driver of risk can be ascertained through group characteristics
Intervention mapping	Less directly useful for intervention mapping	May offer opportunities for direct intervention mapping

The *threshold approach* grouped the final predicted risk scores assigned to each visit by the PREDICT or SLATE model into three pre-set categories: visits with the lowest 50% of scores were assigned a “green” or low-risk category; visits with the middle 40% of scores were assigned a “yellow” or moderate risk category; and visits scoring in the highest 10% of risk scores were assigned a “red” or high-risk category. The origins of the thresholds were chosen by identifying cutoff points that would result in the IIT rate in each bin (red, yellow, green) being twice that of the lower bin (for example, the IIT rate in the red bin is 26% which is twice that of the IIT rate of 13% in the yellow bin). We then considered the visit observed immediately after a “scored” visit (hereafter called “next visit”) and classified this as IIT or not, based on whether the next visit occurred within 28 days of its scheduled date. The proportion of next visits classified as IIT was then estimated for each risk triage category.

The *archetype approach* considered characteristics identified by each model as important predictors of missed visits that could contribute to an individual’s vulnerability to disengagement from care as conceptualized in the framework for reasons for disengagement by Burke et al.^4^ The PREDICT model considered demographic and visit history characteristics available in the routine EMR datasets, while the SLATE model used social, economic, and HIV treatment experience features collected as part of the clinical trial enrolment survey, in addition to demographic and visit history characteristics from the EMR. ART clients were then grouped together into subgroups with a shared set of characteristics, creating distinct sub-population profiles or archetypes.

The archetypes were created by identifying client segments manually by systematically exploring all possible combinations of key risk variables and observing where the highest rates of treatment interruption occurred. Using feature importance tables from the predictive modeling, features were next paired into different configurations. (For example, combining responses to the questions “Has the client disclosed their status?” and “Does the client have enough information to start ART?” yields four configurations: not disclosed and not enough information; have disclosed and not enough information; not disclosed and have enough information; have disclosed and have enough information). We then used these archetypes to isolate subgroups where these factors played a key role in determining the risk of an interruption in treatment. Any logically invalid or very small subgroups were removed to maintain robustness. Finally, the rate of IIT was calculated within each sub-group (or configuration), and all subgroups’ IIT rates were then compared to the whole population’s baseline IIT rate identifying those with the largest positive or negative differences as potential archetypes of interest. This approach ensured that our segmentation was both data-driven and explainable, capturing meaningful risk clusters rather than relying on predefined thresholds.

### Statistical Analysis

For the threshold approach, we first used simple frequencies and proportions to describe the overall number and distribution of visits triaged into each risk category (green, yellow and red groups). We stratified these descriptive statistics by age, gender, and time on ART. Next, we estimated the crude relative risk (RR) and corresponding 95% confidence interval (CI) for IIT at the next visit stratified by the current visit risk triage category, with the green “low risk” group as reference. The analytic approach to the client archetypes was similar. We first described the overall frequency and distribution of visits by clients characterized into each archetype, stratified into age and gender clusters and by time on ART. We then estimated the crude relative risk and corresponding 95% confidence interval of missing a scheduled visit for comparing each archetype to the archetype with the lowest perceived risk of IIT.

## Results

### Characteristics of Study Participants and Model Performance Metrics

The two source data sets are described in Table 2. The original PREDICT model data set utilized routinely collected, anonymized, longitudinal data from >460,000 clients accessing HIV care and treatment during >4.6M visits at public sector treatment sites in Mpumalanga and the Free State between January 2016 and December 2018. The SLATE trials provided a total of 1193 patient records containing 7199 clinic visits in Gauteng. Participant characteristics are summarized in Table 2. We note that the SLATE study population differed substantially from the original PREDICT model dataset by the distribution of stage of HIV care journey. The original PREDICT data set included visits across all stages of care with a median duration on ART of approximately 5 years. SLATE study participants, in contrast, were all enrolled at ART initiation and followed up for a maximum duration of 14 months. Pregnant women were also excluded from the SLATE studies but included in the PREDICT datasets.

**Table 2:** Characteristics of the Study Population

Characteristic	PREDICT Model	SLATE Model
Data source	Routinely collected EMR data	Clinical trial data supplemented with EMR data
Setting	Ehlanzeni District (Mpumalanga) and Thabo Mofutsanyana District (Free State)	City of Johannesburg and Ekurhuleni Districts (Gauteng)
Facility profile (%)		
Urban	52%	67%
Peri-urban	9%	33%
Rural	37%	0%
Missing	2%	0%
Client sample size	463,418 clients	1156 clients
Visit sample size	4,663,816 visits	7199 visits
Current age (median, IQR)	39 years (27–49)	SLATE: 35 years (29–41)
% Female	68%	64%
Prevalent pregnancy	2%	0% (pregnant women were excluded)
Time on ART at entry to cohort (median, IQR)	62 months (30–93 months)	0 months (all newly initiating or re-initiating clients)
Maximum follow up duration	36 months	14 months
Variables with most predictive power in the final model	% Visits attended >3 days late # Times >28 days late # Visits at this facility # VL tests done Months since first visit Months since last visit Current age Day of month of next appointment Viral load value (copies/mm^3^) Day of week of next appointment Visits on regimen Sex (M/F) # Missed months	Time on ART Appointment month Time since last viral load test Viral load value (copies/mm^3^) % Visits attended >3 days late CD4 count at screening Current age Travel time to clinic Total # TB symptoms Year first tested positive # Other people living with the client

**Table 3:** Comparison of SLATE Model Performance Metrics to Original PREDICT Model

Variable	PREDICT Model	SLATE Model with All Variables	SLATE Model Limited to Variables Available in PREDICT
Total sample size	3,264,671 client visits	7199 client visits	7199 client visits
Test set sample size	1,399,145 client visits	1440 client visits	1440 client visits
Accuracy	66% (n = 1,399,145)	63% (n = 1440)	61% (n = 1440)
Sensitivity	61% (n = 146,881)	52% (n = 200)	55% (n = 200)
Specificity	67% (n = 1,252,264)	64% (n = 1240)	61% (n = 1240)
Positive predictive value	18% (n = 503,730 total positive predictions)	19% (n = 544 total positive predictions)	19% (n = 589 total positive predictions)
Negative predictive value	94% (n = 895,415 total negative predictions)	89% (n = 896 total negative predictions)	89% (n = 851 total negative predictions)
AUC	0.688	0.614	0.603

**Abbreviation**: AUC, area under the curve.

The SLATE data set was divided into a training set of 5759 visits by 872 clients and a test set of 1440 visits by 668 clients. In total, 13.5% of visits in the training set and 14.0% visits in the test set were observed to occur >28 days after the scheduled visit date. The algorithm investigated 239 exposure variables in total, including the additional demographic and socioeconomic variables from the SLATE baseline questionnaires. The full set of exposure variables was then reduced to a parsimonious model containing the top 11 exposure features with the most predictive power: time on ART, appointment month, time since last viral load (VL) test, VL test result, proportion of visits attended >3 days late, CD4 count at screening, age, travel time to clinic, total number of TB symptoms, year first tested positive, and number of others living with client in their house.

The SLATE model achieved an accuracy of 63%, specificity of 64%, and negative predictive value of 89% (Table 3), comparable to the original (and much larger) PREDICT dataset which achieved an accuracy of 66%, specificity of 67%, and negative predictive value of 94%.

**Table 4:** Proportion Visits with IIT at Next Scheduled Visit Stratified by Current Visit Risk Triaging Classification and Time on ART (Threshold Approach)

Risk Triaging Classification at Current Visit	All Visits		First Visit After Initiation (n=41,751)		0–6 Months on Art (n=194,469)		7–12 Months on Art (n=129,764)	
	IIT at Next Visit	Relative Risk (95% CI)	IIT at Next Visit	Relative Risk (95% CI)	IIT at Next Visit	Relative Risk (95% CI)	IIT at Next Visit	Relative Risk (95% CI)
*PREDICT model datasets*								
**ALL (n=1,399,145)**	**11%** (n=146,881)		**19%** (7,979/41,751)		**11%** (19,583/194,469)		**10%** (12,970/129,764)	
**GREEN (n=699,573)**	**6%** (n=41,974)	Ref	**13%** (2,714/20,878)	Ref	**6%** (5835/97,245)	Ref	**5%** (3244/64,889)	Ref
**YELLOW** **(n= 559,658)**	**13%** (n=72,756)	RR=2.17 (2.14–2.19)	**21%** (3068/14,609)	RR=1.62 (1.54–1.69)	**12%** (8165/68,044)	RR=2.00 (1.94–2.07)	**11%** (4994/45,404)	RR=2.20 (2.11–2.30)
**RED** **(n = 139,915)**	**26%** (n=36,378)	RR=4.33 (4.28–4.39)	**33%** (2067/6,264)	RR=2.54 (2.42–2.67)	**21%** (6128/29,180)	RR=3.50 (3.38–3.62)	**23%** (4478/19,471)	RR=4.60 (4.41–4.80)
*SLATE model datasets*								
**ALL** **(n=1440)**	**14%** (n=200)		**19%** (34/155)		**15%** (114/791)		N/A	
**GREEN** **(n=720)**	**10%** (n=75)	Ref	**18%** (14/78)	Ref	**10%** (41/396)	Ref		
**YELLOW** **(n= 576)**	**14%** (n=81)	RR=1.41 (1.04–1.89)	**24%** (15/62)	RR=1.35 (0.71–2.58)	**18%** (56/316)	RR=1.79 (1.23–2.60)		
**RED** **(n = 144)**	**31%** (n=44)	RR=3.13 (2.25–4.33)	**33%** (5/15)	RR=1.86 (0.79–4.38)	**22%** (17/79)	RR=2.13 (1.27–3.56)		

**Abbreviation**: Ref, Reference population.

When restricting the SLATE model to the set of variables that were available to the PREDICT model (ie, data from routinely collected medical records only), the performance of SLATE model demonstrated little change from results obtained using all variables available in the SLATE datasets: 61% accuracy, 61% specificity, and 89% negative predictive value. Results using an alternate model building approach (gradient boosting) to the SLATE data are provided in Supplementary Table 1 for comparison. Hereafter, all results from the SLATE datasets refer to the full model using all variables available in the SLATE datasets unless otherwise stated.

### Results for Threshold Approach to Risk Triaging

As explained above, for the threshold approach, results from the predictive models were used to assign a final predictive risk score to every observed visit in the PREDICT and SLATE datasets. These scores were then grouped into centile brackets: the visits with the lowest 50% of scores were assigned a “green” or low-risk category; the middle 40% were assigned a “yellow” or moderate risk category; and visits with the highest 10% of scores were assigned a “red” or high-risk category. We then considered the visit observed immediately after a “scored” visit (hereafter called “next visit”) and classified these as IIT or not based on whether the next visit occurred within 28 days of its scheduled date.

In total, 11% of all visits observed in the PREDICT datasets were classified as IIT (n=146,881 visits). The IIT rate observed for visits in the SLATE datasets was slightly higher, at 14% (n=200 visits; Table 4). Rates of IIT at next visit increased in a linear fashion with the increasing predicted risk threshold categories for the current visit. Compared to green “low risk” visits in the PREDICT datasets, visits classified in a yellow “moderate risk” group were twice as likely to be followed by a treatment interruption (13% IIT at next visit in yellow group versus 6% IIT at next visit for green group; RR=2.17; 95% CI 2.14–2.19), while the red “high risk” triage visits were more than 4 times as likely to be followed by a treatment interruption at next visit compared to visits classified as green (26% IIT at next visit in red group versus 6% IIT at next visit in green group; RR = 4.33; 95% CI 4.28–4.39). Results were similar using the SLATE datasets.

**Figure 1:**
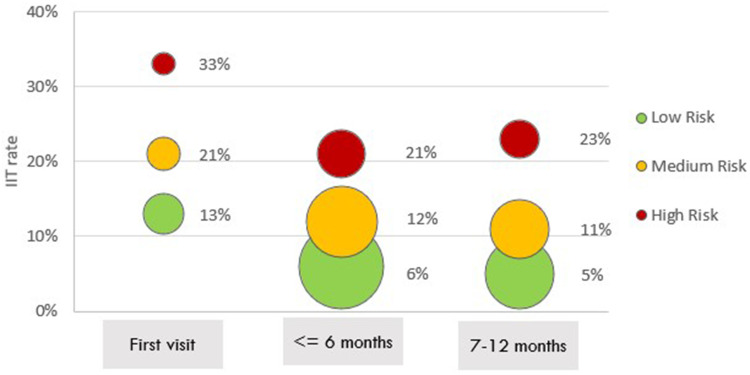
Proportion of visits classified as IIT stratified by risk threshold approach and time on ART (PREDICT model data).

The rate of IIT at next scheduled visit also differed by time on ART for both the PREDICT and SLATE datasets (Figure 1 and Table 4). Risk of IIT at the next scheduled visit after ART initiation was nearly double that of the periods 0–6 months or 7–12 months on ART (19% versus 10%, respectively). Visits that occurred during month 7–12 after ART initiation and were classified as green had the lowest rates of IIT at next scheduled visit (5%), while first visits after initiation that were classified as red were followed by the highest rates of treatment interruption at next scheduled visit (33%). Within the first 6 months on treatment, visits classified as red were more than three times as likely to be followed by a treatment interruption than visits classified as green (RR 3.50; 95% CI 3.38–3.62); during months 7–12 red visits were more than four times as likely to be followed by an IIT at next scheduled visit as were green visits in the same period (5% vs 23%; RR=4.60; 95% CI 4.41–4.80). Models generally performed somewhat better in terms of accuracy, sensitivity, specificity and AUC for the full period 0–6 months on ART compared to predictions made only for the first visit (Supplementary Table 2).

### Results for the Archetype Approach to Triaging

Using characteristics identified by the SLATE model as important predictors of missed visits, we defined archetype profiles across three categories: 1) demographic archetypes based on age and gender; 2) behavioral archetypes based on visit attendance; and 3) social-behavioral archetypes based on client characteristics. In Table 5, we define archetypes within each category, giving each archetype a descriptive label. As the PREDICT datasets did not contain several of the variables needed to define the socio-behavioural archetypes, these are reported for the SLATE datasets only. The SLATE datasets did not observe movement across facilities and so the behavioural archetype “Shopper” is reported for PREDICT only. All other archetypes are reported for both datasets.

**Table 5:** Archetype Approach Definitions

Category and Archetype	Description	Dataset Source	Data Collection Period
***Demographic archetypes***			
Adult female	Female client aged >25 years at date of visit	SLATE and PREDICT, available routinely in EMR	At ART initiation and each follow up visit
Adult male	Male client aged >25 years at date of visit	SLATE and PREDICT, available routinely in EMR	At ART initiation and each follow up visit
Adolescent girls and young women	Female client aged between 15 and 25 years at date of visit	SLATE and PREDICT, available routinely in EMR	At ART initiation and each follow up visit
Adolescent boys and young men	Male client aged between 15 and 25 years at date of visit	SLATE and PREDICT, available routinely in EMR	At ART initiation and each follow up visit
***Behavioral archetypes***			
Prompt and loyal	Visit attended on time and only attended care at this facility	SLATE and PREDICT, available routinely in EMR	At each follow up visit
Late twice	The previous two visits were attended after the scheduled appointment date	SLATE and PREDICT, available routinely in EMR	At each follow up visit
Shopper, no number	Has attended at other facilities and no phone number on file	SLATE and PREDICT, available routinely in EMR	At each follow up visit
Returning after disengagement	At least one prior visit was attended >28 days late	SLATE and PREDICT, available routinely in EMR	At re-initiation visit
***Socio-behavioral archetypes***			
Super green	Punctual visit attendance, does not live alone	SLATE, not available routinely in EMR	At ART initiation and each follow up visit
Employed youth at payday	Age 18–29, identify as employed AND next visit scheduled <7 days from payday	SLATE, not available routinely in EMR	At ART initiation and each follow up visit
Prior test and prompt	Has a history of HIV testing (before testing positive) AND regularly prompt for visits	SLATE and PREDICT, available routinely in EMR	At ART initiation and each follow up visit
Lone ranger	Lives alone or with 1 other person AND lives more than 20 mins away	SLATE, not available routinely in EMR	At ART initiation
Unexpected and unsupported	Was not planning to test for HIV today AND lives alone/with 1 other person	SLATE, not available routinely in EMR	At ART initiation
Disillusioned disclosers	Identifies as having HIV info, has disclosed, lives alone/with 1 other person	SLATE, not available routinely in EMR	At ART initiation
Live close, always late	Lives <20 mins from clinic but is also regularly late for appointments	SLATE, not available routinely in EMR	At ART initiation and each follow up visit
Prepared and late	Prepared to start ART today, has tested before but is late to appointments	SLATE, not available routinely in EMR	At ART initiation and each follow up visit

Adult females comprised the largest demographic group in both the SLATE and PREDICT datasets (57%) and between 10% and 13% of visits made by adult females were classified as IIT (Table 6). Adult males made up nearly a third of clients in both datasets, with 12–14% of visits made by adult males classified as IIT. Few adolescent or young men and women (8%) were observed in the SLATE datasets, as the trials enrolled participants >18 years of age only. Despite being one of the smallest population groups, adolescent girls and young women (AGYW) demonstrated the highest rates of IIT across the demographic archetypes (15% in SLATE and 16% in PREDICT) and were more likely to have a treatment interruption compared to adult women in both the PREDICT (RR=1.52; 95% CI 1.49–1.55) and SLATE datasets (1.15; 95% CI 0.91–1.46).

**Table 6:** Proportion of Visits Classified as IIT Stratified by Archetype Triaging Approach

Archetypes	Slate Data (N=7199 Visits)			Predict Data (N=925,639 Visits)**		
	Visits	IIT (n, %)	RR (95% CI)*	Visits	IIT (n, %)	RR (95% CI)*
**Demographic archetypes (variables available in both SLATE and PREDICT models)**						
Adult females	4141 (57%)	555 (13%)	Ref	572,154 (57%)	58,271 (10%)	Ref
AGYW	434 (6%)	67 (15%)	1.15 (0.91–1.46)	70,045 (7%)	10,864 (16%)	1.52 (1.49–1.55)
ABYM	146 (2%)	19 (13%)	0.97 (0.63–1.49)	10,444 (10%)	1304 (13%)	1.23 (1.17–1.29)
Adult males	2478 (34%)	893 (14%)	1.02 (0.9–1.14)	272,996 (27%)	31,279 (12%)	1.20 (1.19–0.21)
**Behavioral archetypes (variables available in both SLATE and PREDICT models)**						
Prompt and loyal	1552 (22%)	227 (15%)	Ref	652,595 (65%)	59,099 (9%)	Ref
Late twice	854 (12%)	155 (18%)	1.24 (1.03–1.50)	97,986 (10%)	15,932 (16%)	1.80 (1.76–1.83)
Shopper no-number	N/A			68,087 (7%)	11,360 (17%)	1.91 (1.87–1.94)
Returning after disengagement	861 (12%)	169 (20%)	1.34 (1.12–1.61)	37,404 (4%)	9280 (25%)	2.74 (2.69–2.80)
**Socio-behavioral archetypes (variables available in SLATE model only)**						
Super green	2313 (32%)	239 (10%)	Ref			
Employed youth at payday	301 (4%)	35 (12%)	1.13 (0.81–1.57)			
Prior test and prompt	1789 (25%)	228 (13%)	1.23 (1.04–1.46)			
Lone ranger	1478 (21%)	221 (15%)	1.45 (1.22–1.72)			
Unexpected and unsupported	817 (11%)	120 (15%)	1.42 (1.16–1.74)			
Disillusioned disclosers	1194 (17%)	184 (15%)	1.49 (1.25–1.78)			
Live close but always late	986 (14%)	167 (17%)	1.64 (1.36–1.97)			
Prepared and late	501 (7%)	93 (19%)	1.80 (1.44–2.24)			

**Notes**: *RR = Relative risk, reported with 95% confidence interval. **Data restricted to visits within the first 6 months on ART.

**Abbreviations**: AGYW, adolescent girls and young women; ABYM, adolescent boys and young men.

Several of the identified behavioral archetypes were also at increased risk of IIT at next visit compared to their reference groups. Clients who had been late for at least two prior visits were more likely to have an IIT at next visit compared to all adult women (Table 6) in both datasets. Those who were returning after previously disengaging from care were at the highest risk of IIT compared to adult females (RR = 2.44; 95% CI 2.40–2.48 in PREDICT and RR=1.46; 95% CI 1.25–1.71 in SLATE). When combining social and behavioural characteristics (SLATE data only), the client archetypes least likely to have an IIT at next visit were those who attended prior visits on time, were young and employed, and had a history of previous HIV testing. Those who lived alone, did not have a treatment supporter, or were not expecting to start HIV treatment at initiation were at increased risk of having a treatment interruption. Compared to those who attend visits on time and do not live alone, youth who reported being employed and had a visit scheduled within 7 days of payday were at a somewhat increased risk of a subsequent treatment interruption (12% IIT; RR = 1.13; 95% CI 0.81–1.57).

We also stratified the behavioural and socio-behavioural archetypes by age and gender and noted varying risk for different substrata of the population (Table 7). In particular, we noted that the behavioural elements (visit attendance) of the archetypes tended to drive the risk of treatment interruption more consistently than the basic demographic elements. For example, adolescent boys and young men who attended visits on time experienced one of the lowest rates of treatment interruption (10%, PREDICT datasets and 7% SLATE datasets), while adolescent boys and young men who had returned after previously disengaging in care were the group with the highest rates of subsequent treatment interruption (31%, PREDICT datasets and 40% SLATE datasets). Similarly, adolescent girls and young women returning after a period of disengagement were 3.5 times more likely to have a treatment interruption when compared to adult females (RR=3.50; 95% CI 3.32–3.68; PREDICT datasets). In fact, even a visit history of attending late twice among adolescent girls and young women was associated with a subsequent treatment interruption (21% IIT, RR=2.50 (95% CI 2.39–2.61) in PREDICT data and 26% IIT, RR=1.93 (95% CI 1.23–3.05) in SLATE datasets) compared to all adult females. Other socio-behavioural archetypes associated with increases in risk for subsequent treatment interruption regardless of demographic profile included archetypes characterized by limited or no social support at home and living alone and/or at a far distance from the clinic.

**Table 7:** Proportion of Visits Classified as IIT Stratified by Archetype Triaging Approach and Demographics (PREDICT and SLATE Data)

Model	Visits (n, %)	IIT%	RR	95% CI
Predict Data Archetypes (N=925,639 Visits)*				
**All adult females (reference group)**	***572,154 (57%)***	***10%***	***Reference***	** **
*Adult females prompt and loyal*	384 316 (39%)	8%	0.83	0.82–0.84
*Adult males prompt and loyal*	173 750 (17%)	9%	0.91	0.89–0.92
*ABYM prompt and loyal*	6511 (1%)	10%	0.96	0.89–1.03
*AGYW prompt and loyal*	43,938 (4%)	13%	1.29	1.26–1.32
*Adult females late twice*	51,244 (5%)	15%	1.47	1.44–1.51
*Adult females shopper no-number*	40,627 (4%)	16%	1.56	1.53–1.60
*Adult males late twice*	30,554 (3%)	17%	1.65	1.60–1.69
*Adult males shopper no-number*	18,945 (2%)	17%	1.70	1.65–1.76
*ABYM shopper no-number*	667 (0%)	18%	1.78	1.52–2.09
*ABYM late twice*	1190 (0%)	18%	1.79	1.59–2.02
*AGYW late twice*	7371 (1%)	21%	2.07	1.98–2.16
*AGYW shopper no-number*	4194 (0%)	22%	2.11	1.99–2.24
*Adult females returning after disengagement*	19,784 (2%)	23%	2.29	2.23–2.35
*Adult males returning after disengagement*	9874 (1%)	26%	2.56	2.47–2.65
*AGYW returning after disengagement*	3639 (0%)	30%	2.90	2.75–3.05
*ABYM returning after disengagement*	426 (0%)	31%	3.07	2.66–3.53
**Slate Data Archetypes (N=7,199 VISITS)**				
**All adult females (reference group)**	***4,141 (57%)***	***13%***	***Reference***	** **
*AGYW prior test and prompt*	52 (1%)	6%	0.43	0.14–1.30
*AGYW super green*	49 (1%)	6%	0.46	0.15–1.37
*ABYM prompt and loyal*	30 (0,5%)	7%	0.50	0.13–1.90
*Adult females super green*	1418 (20%)	10%	0.67	0.56–0.80
*Adult males super green*	698 (10%)	11%	0.79	0.63–0.99
*AGYW lone ranger*	82 (1%)	11%	0.82	0.44–1.52
*ABYM super green*	148 (2%)	12%	0.91	0.59–1.41
*Adult males unexpected and unsupported*	370 (5%)	12%	0.91	0.68–1.21
*Adult males prior test and prompt*	636 (9%)	12%	0.92	0.73–1.14
*Adult females prior test and prompt*	1012 (14%)	13%	0.97	0.81–1.17
*AGYW unexpected and unsupported*	60 (1%)	13%	1.00	0.52–1.91
*Adult males disillusioned disclosers*	453 (6%)	14%	1.05	0.83–1.34
*Adult males lone ranger*	533 (7%)	15%	1.09	0.88–1.36
*Adult males prompt and loyal*	529 (7%)	15%	1.11	0.90–1.39
*Adult females prompt and loyal*	875 (12%)	15%	1.11	0.92–1.33
*Adult females live close but always late*	521 (7%)	15%	1.15	0.93–1.44
*ABYM prior test and prompt*	89 (1%)	16%	1.17	0.72–1.91
*Adult females lone ranger*	856 (12%)	15%	1.19	0.99–1.42
*Adult females disillusioned disclosers*	720 (10%)	15%	1.19	0.98–1.43
*AGYW prompt and loyal*	118 (2%)	16%	1.20	0.79–1.82
*Adult males live close but always late*	378 (5%)	17%	1.28	1.01–1.62
*Adult females late twice*	489 (7%)	17%	1.30	1.04–1.61
*Adult females unexpected and unsupported*	378 (5%)	17%	1.32	1.04–1.67
*Adult females prepared and late*	338 (5%)	18%	1.36	1.07–1.74
*Adult male late twice*	292 (4%)	19%	1.38	1.07–1.78
*Adult males returning after disengagement*	291 (4%)	19%	1.44	1.12–1.84
*Adult females returning after disengagement*	509 (7%)	19%	1.49	1.22–1.82
*Adult males prepared and late*	131 (2%)	21%	1.54	1.09–2.17
*AGYW returning after disengagement*	41 (1%)	22%	1.64	0.92–2.93
*ABYM unexpected and unsupported*	9 (0%)	22%	1.66	0.49–5.64
*AGYW live close but always late*	59 (1%)	24%	1.77	1.11–2.82
*AGYW late twice*	54 (1%)	26%	1.93	1.23–3.05
*ABYM late twice*	19 (0%)	26%	1.96	0.92–4.18
*ABYM live close but always late*	28 (0%)	32%	2.40	1.39–4.13
*ABYM returning after disengagement*	20 (0%)	40%	2.99	1.73–5.14
*ABYM lone ranger*	7 (0%)	43%	3.20	1.36–7.55

**Note**: *Data restricted to visits within the first 6 months on ART.

**Abbreviations**: RR, Relative risk; 95% CI, 95% confidence interval; AGYW, adolescent girls and young women; ABYM, adolescent boys and young men.

**Figure 2:**
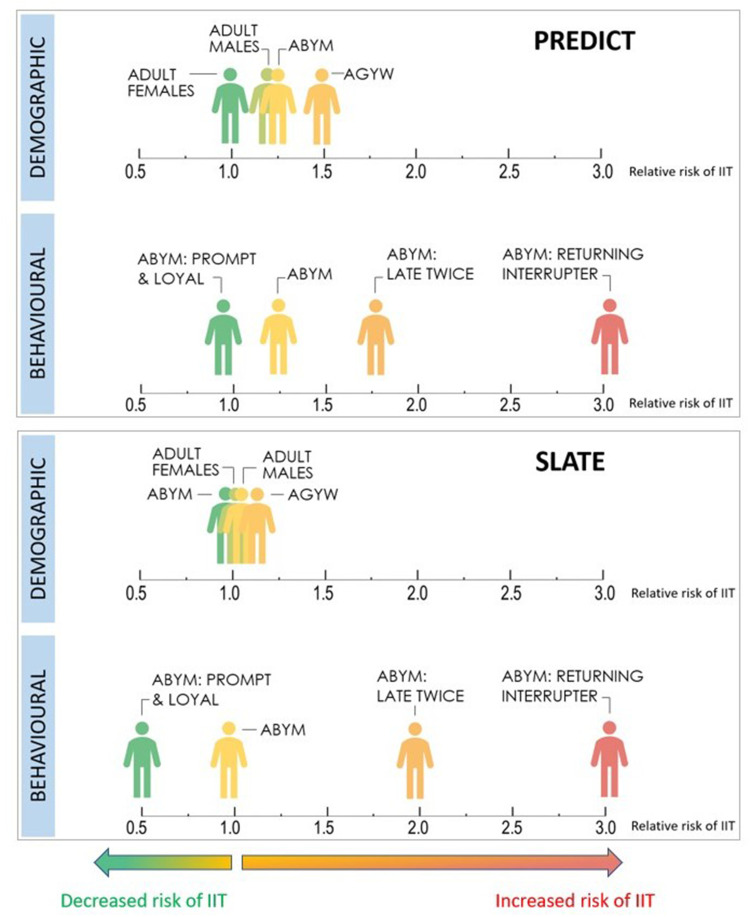
Relative risk of interruption in treatment (IIT) at next scheduled visit stratified by risk archetype and demographic strata.

**Abbreviations**: ABYM, adolescent boys and young men; AGYW, adolescent girls and young women.

The development of the behavioural archetypes provided a more granular characterization of risk within each demographic stratum compared to a single risk estimate for any one demographic group. Figure 2 offers a visual depiction of how the point estimates for risk of treatment interruption vary when stratifying risk using demographic characteristics only compared to stratifying risk by combined demographic and behavioural characteristics. For both PREDICT and SLATE datasets, when the risk of IIT is stratified by demographic characteristics only (gender and age), we find estimates of risk tend to cluster close together. In the SLATE datasets, for example, the risk of IIT ranged from a relative risk of 0.97 (95% CI 0.63–1.49) for adolescent boys and young men to a relative risk of 1.15 (95% CI 0.91–1.46) among adolescent girls and young women; suggesting adolescent boys to be at similar risk for IIT compared to adult women. However, when the behavioral archetypes are considered within a singular demographic stratum (in this case, restricting to adolescent boys and young men), the point estimates for the relative risk of treatment interruption at next scheduled visit spans a much wider range and subgroups with varying risk of IIT are revealed, characterized largely by prior visit attendance. The behavioural archetypes indicate that adolescent boys and young men who have attended clinic visits on time are at low risk of IIT at next visit compared to adult females, while those who have attended visits late at least twice in the past are twice as likely to experience treatment interruption (RR=2.16; 95% CI 1.91–2.44; PREDICT datasets) and those who have previously disengaged from care are three times as likely to interrupt treatment at next scheduled visit (RR=3.07; 95% CI 2.66–3.53; PREDICT datasets).

## Discussion

As HIV service delivery models expand and evolve, ensuring sustained client retention after treatment initiation remains a key priority. Many interventions to address disengagement from care are either applied universally to all clients engaged in treatment programs or reactively after clients have disengaged from care;^35^,^36^ both scenarios utilize resources that do little to improve program outcomes. In this paper, we present a novel application of machine learning and predictive algorithms to develop approaches that identify clients who are at heightened risk of disengagement before they experience interruptions in care or disengage entirely. Both of our models, if used in the course of a routine clinic visit, would allow healthcare providers to target interventions substantially more accurately than is currently possible, potentially improving retention among those at risk for disengagement if suitable interventions to address underlying drivers of risk are available as part of routine care.

Both of the approaches we report were successful, though in different ways. We note first that the original PREDICT model is reproduced well in the SLATE datasets. Despite the differences in population, geography, and sample size, the results were very similar in terms of model performance metrics between the PREDICT (Accuracy 66%, AUC 0.69, NPV 94%) and SLATE models (Accuracy 63%, AUC 0.61 and NPV 89%). Across varying approaches and classification algorithms, the models were able to consistently predict approximately two in three visits classified as treatment interruptions. The performance metrics of other machine learning approaches applied to HIV clinical data have varied, with accuracy ranging from 70% to 91% across 8 models predicting loss from care in Nigeria.^25^ A Kenyan model identifying suppressed VL^26^ reported an AUC of 0.56, while another from Ethiopia reported an AUC of 0.99.^19^

The threshold approach is also useful for categorizing client groups at functional risk levels and offers an opportunity to triage clients for different intensity interventions. For example, our results confirm that rates of IIT are high for some clients during the first 6 months but are not universally so for all clients. The threshold approach to triaging was able to identify sub-groups of early ART clients most at risk for a treatment interruption (red group) and others at low risk (green group). This approach that can be readily interpreted and easily adapted into a point of care questionnaire that asks the client questions related to the most predictive features of the risk model, with scores allocated to each response and a tally score at the end which would identify the overall risk group (high, medium or low) for each client at point-of-care. This could allow for triaging of clients at the facility level into high- or low-intensity models of service delivery before clients are eligible for existing differentiated service delivery models. Given that low-risk visits comprised half of all visits and were associated with very low rates of IIT (6%), shifting clinician time and facility resources to higher risk clients could translate into important gains in efficiency without compromising quality of care for low-risk groups.

The second approach, the archetype approach to risk triaging confirmed several important points. First, patterns of visit attendance are key in identifying the risk of IIT in both directions, regardless of demographic sub-group. Of the three socio-behavioural risk groups with the lowest rates of IIT, two were characterized by on-time visit attendance (“super green” and “prior test and prompt”). In contrast, the three archetypes at highest risk of IIT (“returning after disengagement”, “live close but always late” and “prepared and late”) were all characterized by a history of late appointments or prior disengagement in care. This suggests that client behavior, as revealed by visit attendance, tends to be consistent and may present opportunities to intervene prior to disengagement among those who are at higher risk. It also allows providers to identify low-risk groups who not only represent an important share of clients attending facility visits (32% of visits were among clients characterized as “super green”) but could potentially be safely managed with lower intensity models of care immediately after ART initiation, allowing for reallocation of time and resources to groups identified as priority risk groups. The use of behavioural archetypes allows for a more granular and detailed characterization of risk within a particular demographic profile (Figure 2 and Supplementary Table 3). When the risk of IIT is estimated for each of the behavioral archetypes within a singular demographic stratum, key sub-groups at increased risk of IIT are revealed; again, characterized largely by prior visit attendance. In this way, adolescent boys and young men simultaneously represent both the group at lowest risk of IIT at next visit (ABYM who have attended visits at the originating facility on time) as well as the group at nearly the highest risk of IIT (ABYM returning after previously disengaging from care). The behavioural archetype approach allows for the identification of subgroups that have similar demographic characteristics but likely require quite different intervention strategies to support continuous engagement in care. As the information required to profile a client into a behavioural archetype is readily available at point-of-care, this approach may offer the potential to tailor interventions to specific groups in a more targeted way than has been available previously.

Where social and behavioural data are available, utilizing the archetype approach can also contribute to understanding not only particular client subgroups that are at risk of treatment interruption but also insight into the mechanisms underlying the increased risk. For example, when the socio-behavioural archetypes are stratified by demographic profiles (Table 7) we see a higher risk of IIT for AGYW who are classified as disillusioned disclosers and ABYM meeting the lone ranger archetype. Both of these archetypes are characterized by living alone, which suggests that young persons living with HIV may be vulnerable to a lack of social support as they navigate their HIV care journey. This knowledge could inform service delivery models providing differentiated care to this age group. In addition, we noted that while youth are generally at higher risk of treatment interruption, the subgroup of youth who reported being employed and had a visit scheduled within 7 days of payday were an archetype with one of the lowest risk of a subsequent treatment interruption (RR=1.13, 95% CI 0.81–1.57; Table 6). This suggests that scheduling of clinic visits may be important for successful attendance among those with work commitments or where access to money for transport is key.

**Figure 3:**
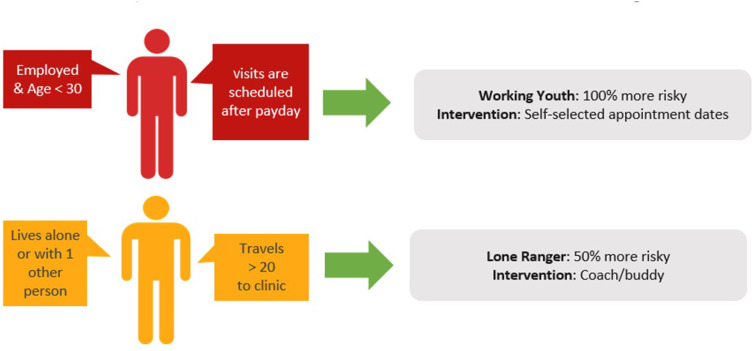
Schematic of intervention mapping guided by behavioural archetype.

Finally, where the archetype approach to risk triaging provides insight into underlying drivers of the risk of treatment interruption, it also creates the opportunity to map appropriate interventions to groups of ART clients most likely to benefit from them (Figure 3). For example, archetypes characterized by a lack of social support might be offered a treatment buddy or coach to assist them in establishing care during the early treatment period. Alternatively, a health worker might consider offering the choice of appointment scheduling to the employed youth – those who struggle to attend near payday because of work commitments might prefer a visit date earlier in the month, while another youth who needs their wages for transport money may prefer a visit scheduled shortly after payday. Used in this way, risk triaging offers an opportunity to optimize the impact of retention interventions by offering them to those among whom such interventions are most likely to have a positive impact on visit attendance while also reducing unnecessary resource expenditure by not offering the same interventions to clients who may neither want nor need them.

There may also be benefit in considering how the threshold and behavioural approaches could be combined to maximise service delivery efficiency. For example, a client’s clinic encounter could start with the threshold approach, in which clients at risk of disengagement from care could be differentiated from those who are unlikely to need further assistance at that time (*Who is currently at risk?*). Once this is established, the archetype approach could be implemented among clients at risk of disengagement to understand the factors driving their current risk state (*Why is this client at risk at this time? What can be done about this?)*. This would allow providers to focus the time and resources needed on clients most likely to benefit from additional support or modification to their service delivery options while not adding additional clinic visit time for clients at low risk.

In addition, consideration of how automation of these approached could be achieved is worth noting. Practical use of these risk-triaging approaches is possible through the implementation of software in the facility as standalone applications or as modules within existing software. The research team has piloted two approaches to the implementation of the threshold approach – as a standalone application that adds supplementary information (risk predictions) to routinely used spreadsheets from the EMR and as a module in an implementing partner’s case management scheduling application. The archetype approach could be similarly implemented by assigning archetypes to visits on existing patient lists exported from EMR systems. Automation of these approaches via simple logic (for example, are patients male and under 25?) or via machine learning requires routine logging and tracking of dynamic changes in the population and algorithm performance. In the case that changes have occurred, the software needs to be readily updated or include capabilities to automatically retrain the algorithm and deploy new logic.

Our results should be interpreted in light of the limitations in both the data sources used in this analysis and our approaches. Importantly, both data sources only observed clinic visits at the originating site. Clients may have attended HIV care at another facility but not been observed in the analytic datasets presented here; such visits may be misclassified as “not attended”. This is a common drawback to studies that rely on unlinked clinic records, as we did in both our analytic approaches (threshold and archetype).^26^ To the extent that this happened, our results will overestimate proportions of clinic visits not attended. We note that the generalizability of the SLATE data results could be limited by the fact that the data were generated as part of two relatively small clinical trials in a single province. All exposure data, however, were collected in the study baseline questionnaire and follow-up of study participants occurred passively through review of routine electronic medical records that are used across all public sector HIV care facilities and thus were not subject to clinical trial conditions. In addition, during the evaluation of the models for the threshold approach, we utilised metrics other than accuracy, namely ROC-AUC, precision, and recall to give a rounded view of model performance and avoid misleading evaluations. Understanding the distribution of false positives and false negatives provides insight into the model’s real-world performance and fairness. However, the results from the SLATE dataset, particularly those related to the social-behavioural archetypes that used data not routinely collected in the EMR, are moderately scalable within similar communities in Gauteng province given the number of observations and the sampling bias applied during the study. Transferability of the threshold approach has been demonstrated in multiple contexts, including in south Africa’s Gauteng and North West provinces^30^ and also Nigeria (unpublished data, Ijaiya et al).

The archetype results reflect several other limitations as well. Development of the client archetype approach is limited to the variables that were collected in the SLATE trials; other important archetypes may exist that we are unable to describe due to this limitation. The archetyping approach itself may be limited by the need for variables that are not currently routinely collected, though information such as whether a client lives alone or not could easily be added to routine data collection forms if it is proved of sufficient value. Finally, the generalizability of the archetype approach in particular may be limited to South Africa, as it relies on social and behavioral variables that vary by geographic location or culture. Similar to the threshold approach, though, we note that conclusions drawn from the demographic and behavioural archetypes may be considered scalable given the similar findings across the PREDICT and SLATE datasets but also within behavioural archetypes described previously.^37^

## Conclusions

Despite the limited number of implemented tools and some potential limitations, our results have several important implications for HIV service delivery. The South African National ART program seems particularly well-positioned to benefit from these approaches to identifying clients at risk of specific outcomes. As noted above, treatment interruptions, disengagement from care, and reengagement after interruptions are very common occurrences, making effective targeting of interventions especially important. The threshold risk triaging approach allows for identifying clients at risk of treatment interruptions, while the archetype approach identifies underlying obstacles to visit adherence and could point to specific interventions relevant to different types of patients. Potential health system effects of successful application of predictive modelling thus include provision of more relevant service delivery to fewer clients,^25^,^38^ greatly increasing health system efficiency and allowing resources to be targeted to clients who can benefit most. Importantly, a successful risk prediction tool will allow providers to identify those at risk before harmful effects occur. This approach thus has the potential to prevent disengagement from care, rather than simply responding to it after the fact. Future work should address changes in risk states identified through these triaging approaches and the implications for long-term retention on ART. Finally, beyond improving the accuracy of risk prediction, our results represent an important step in introducing the results of machine learning and predictive analytic risk profiling into a routine practice setting. A simple tool for healthcare providers to utilize at the point of care, before clients experience negative outcomes, may be feasible using the characteristics found to be most predictive of future ITT in this study.

## Data Availability

All data results produced in the present study are contained in the manuscript and Supplementary Material. Source data for the SLATE model are available online at Boston University’s data repository. Source data for the PREDICT models are owned by the South African Government and were used under license for the current study. Access to these data was provided by the South African National Department of Health through an agreement with Right to care and is subject to restrictions owing to privacy and ethics policies set by the South African Government, so they are not publicly available. Requests to access these should be directed to pedro.pisa@righttocare.org.
